# Barriers to helicopter emergency medical services in a Haze-Prone, mountainous region of Northern Thailand

**DOI:** 10.1186/s13049-025-01498-w

**Published:** 2025-11-13

**Authors:** Krongkarn Sutham, Prasan Piamanan, Boonyarit Kamthip, Weerapont Kaewpaengchan, Nattikarn Meelarp, Wachira Wongtanasarasin, Borwon Wittayachamnankul

**Affiliations:** 1 Department of Emergency Medicine, Faculty of Medicine, Chiang Mai University, Chiang Mai, Thailand; 2 Department of Emergency Medicine, Nakornping Hospital, Chiang Mai University, Chiang Mai, Thailand; 3 Sriphat Medical Center, Faculty of Medicine, Chiang Mai University, Chiang Mai, Thailand

**Keywords:** Air ambulances, Emergency medical services, Helicopters, Air pollution, Southeast asia

## Abstract

**Study objective:**

To identify factors associated with the mission outcome (transport completed vs. not transported) of air medical transport (AMT), with particular attention to environmental and operational constraints such as haze-related air pollution.

**Methods:**

We conducted a retrospective analysis of all requests to the Northern Thailand Sky Doctor program (SkyDoc) from Jan 1, 2020, to Sept 30, 2024. Patient demographics, mission characteristics, environmental conditions, and operational variables were extracted from program records and merged with daily PM2.5 and PM10 concentrations from the national air quality monitoring network. The primary outcome was mission outcome, defined as transport completed versus not transported. We analyzed factors associated with non-transport.

**Results:**

Among 656 mission requests, 412 (62.8%) were transport and 244 (37.2%) were not transported. Patients were predominantly male (57.2%) with a median age of 49 years (IQR 28–67). The most frequent diagnostic categories were ST-elevation myocardial infarction (32.3%), major trauma (21.2%), and stroke (11.7%). Non-transport was most often due to medical contraindications (25.8%), aircraft or landing-zone unavailability (23.8%), adverse weather (18.9%), and haze-related air pollution (12.7%). Seasonal peaks in not transport were observed from February through April, coinciding with regional surges in particulate matter. In multivariable analysis, twilight-hour requests (16:00–20:00) were associated with non-transport (adjusted OR 0.29; 95% CI 0.13–0.67; *p* < 0.01), whereas secondary missions were more likely to be transport than primary missions (adjusted OR 3.51; 1.47–8.40; *p* < 0.01). Higher PM2.5 concentrations were independently associated with non-transport (adjusted OR 0.99; 0.97–0.99; *p* = 0.018). PM10 was not associated with outcomes.

**Conclusion:**

AMT in northern Thailand is constrained by both medical and environmental factors. Twilight-hour requests, mission type, and PM2.5 concentrations independently influenced mission completion. These findings highlight the need for haze-adapted flight protocols, enhanced forecasting, and system-level strategies to safeguard patient access to aeromedical care during seasonal pollution surges.

**Supplementary Information:**

The online version contains supplementary material available at 10.1186/s13049-025-01498-w.

## Key points

**What is already known on this topic:**

Air Medical Transport (AMT) services are essential for timely access to advanced care in remote and mountainous regions. Operational limitations such as weather, geography, and patient condition are recognized barriers, but the impact of environmental air pollution on aeromedical transport has been poorly studied.

**What this study adds:**

In northern Thailand, we found that twilight-hour requests, primary missions, and elevated PM2.5 concentrations were independently associated with air medical non-transport. Seasonal haze surges created critical access barriers during periods of heightened clinical demand.

**How this is relevant to clinical practice:**

These findings highlight the need for haze-adapted flight protocols, improved forecasting systems, and policy interventions to strengthen AMT resilience in environments affected by air pollution and complex terrain.

**Supplementary Information:**

The online version contains supplementary material available at 10.1186/s13049-025-01498-w.

## Introduction

Air Medical Transport (AMT) is a crucial component of prehospital care, particularly in rural and remote areas where ground transportation may be delayed or unavailable [1]. AMT reduces time to definitive care and improves outcomes in time-sensitive emergencies such as trauma, acute coronary syndromes, and stroke [2–4]. In Thailand, the Sky Doctor program delivers this essential service across various regions, including the mountainous areas of the north. The team oversees AMT operations in the northern region, coordinating both secondary missions (inter-hospital transfers) and primary missions (scene responses) using helicopter emergency medical services (HEMS) from government sectors. These missions often originate from rural health stations or are activated by village health volunteers, serving as a vital link to higher-level care [5].

Despite the essential role of air medical services, multiple barriers routinely limit the feasibility of flights [6]. Globally, these challenges can be categorized into four broad areas. First, resource constraints, such as the limited availability of aircraft and trained personnel, often reduce system responsiveness [7, 8]. Second, infrastructure barriers including inadequate helipad networks and difficult terrain can delay or prevent access to remote locations [9, 10]. Third, environmental and meteorological factors, including extreme weather [11], snowstorms [12], and typhoons [13], frequently lead to flight cancellations or delays, as reported in regions such as Alaska, India, and Taiwan. In Northern Thailand, seasonal haze often suppresses HEMS requests, as flight infeasibility is presumed, and patients are managed instead by ground transfer. Lastly, operational inefficiencies, including communication breakdowns and fragmented coordination between agencies, further complicate timely deployment [7].

In high-income settings, these challenges are often mitigated by robust systems and dedicated fleets. In contrast, Southeast Asia, Thailand specifically, faces unique regional challenges compounded by limited health system resources, fragmented inter-agency coordination, and an underdeveloped data infrastructure. Yet, there has been no systematic investigation into the factors contributing to AMT not completed in Southeast Asia. A clearer understanding of these barriers could inform policy and operational improvements, reduce preventable non-transport, and enhance equity in emergency care delivery. This study aims to identify factors associated with non-transport among requests for AMT in Northern Thailand.

## Methods

### Study design and setting

We conducted a retrospective observational study to evaluate factors associated with not-transport AMT requests in Region 1 of Northern Thailand, which encompasses mountainous provinces such as Chiang Mai, Chiang Rai, Mae Hong Son, Nan, and their surrounding areas. The study period spanned from January 1, 2020, to September 30, 2024, using data from the regional Sky Doctor program, an established AMT system. This study was conducted using de-identified retrospective data. Ethical approval by the Research Ethics Committee of the Faculty of Medicine, Chiang Mai University, granted a waiver of informed consent (registration number EME-2568-0410). All methods were performed in accordance with relevant guidelines and regulations, including the principles of the Declaration of Helsinki.

### Study population and data source

All AMT requests recorded through the SkyDoc system during the study period were reviewed. Each record included patient demographics, diagnosis, time and date of request, referring and receiving hospitals, transport outcome (whether transported or not), and stated reasons for non-transport (denial reasons) when applicable. All 656 mission requests recorded during the study period met inclusion criteria and were analyzed.

### Variable definitions

The age group was classified as follows: Infant (< 1 year), Pediatric (1–15 years), Adult (> 15 years), or Unknown. The province of origin was inferred from referring hospital names.

Air Pollution Data, which were defined as daily PM2.5 and PM10 values, were retrieved from the Pollution Control Department, Thailand, via https://air4thai.pcd.go.th, and matched to each case by province and date of request. Monitoring station locations were geospatially matched to patient provinces. For each case, the average PM2.5 and PM10 value on the date and location of the request was linked to the patient record. When direct measurements were unavailable, values were imputed from the nearest available station on the same day.

Non-transport cases reasons were categorized based on a structured text review. Each AMT request was evaluated by an on-call aviation medical director (AVD) who is an emergency medicine specialist with expertise in prehospital care. The AVD rendered a decision within 40 min using the CSTAT framework (Contraindications, Safety considerations, Transport team availability, Aircraft status and route feasibility, and Time efficiency compared to ground transport). In this framework, ‘C’ included considerations such as COVID-19 risk, while ‘S’ encompassed weather and air pollution risks. This structured approach, which serves as the standard protocol in this region, ensures consistent, timely, and safety-oriented decision-making.

The reasons for non-transport were extracted from structured text and categorized as follows: Haze-related (high PM2.5/PM10 and visibility limitations), Weather-related (non-haze) (e.g., rain, storm), Lack of aircraft or landing constraints, Medical contraindication, Inappropriate timing (twilight hours (16:00–20:00), crew duty-time limits, overlapping missions, or local curfew restrictions), No time benefit over ground transport, Poor prognosis, Medical team issue and Unspecified. Each case with a non-transport outcome was assigned to a category based on manual review of the reason text. The primary outcome was mission outcome, defined as transport completed versus not transported.

### Statistical analysis

Descriptive statistics summarized patient characteristics. Categorical variables were summarized as counts (%) and continuous variables as medians (IQR). Univariate comparisons used chi-square or Fisher’s exact tests, as appropriate. Variables with *p* < 0.10 or strong clinical plausibility entered a multivariable logistic regression model. Odds ratios (ORs) and 95% confidence intervals (CIs) were reported. In the primary analyses, records were retained and missing values were coded as an explicit ‘Unknown’ category where feasible. To assess robustness, we also conducted sensitivity analyses, which yielded results consistent in direction and magnitude with the primary model. All analyses were performed using Python (Pandas, NumPy, Statsmodels, Seaborn, Matplotlib). A *p*-value < 0.05 was considered statistically significant.

## Results

From January 1, 2020, to September 30, 2024, 656 AMT requests were logged by the SkyDoc program; 412 (62.8%) were transport and 244 (37.2%) were non-transported. No cases were excluded (Table 1) Patients were predominantly male (375, 57.2%) with a median age of 49 years (IQR 28–67). Most missions were secondary inter-hospital transfers (524, 79.9%). The most frequent diagnostic categories were ST-elevation myocardial infarction (212, 32.3%), major trauma (139, 21.2%), and stroke (77, 11.7%). The two most common origin provinces were Chiang Mai (377, 57.5%) and Mae Hong Son (265, 40.4%). Survival data are based on available cases only (> 30% missing).

**Table 1:** Demographic data

Variable	Total Case (*N* = 656)	Not Transported (*N* = 244)	Transported (*N* = 412)	*p*-value
**Gender -** *N* **(%)**				< 0.01*
Male	375 (57.2)	131 (53.7)	244 (59.2)	
Female	197 (30.0)	67 (27.5)	130 (31.6)	
Unknown	84 (12.8)	46 (18.9)	38 (9.2)	
**Median age (IQR)**	49 (28–67)	49 (27–65)	49 (28–66)	0.23
**Age group -** *N* **(%)**				< 0.01*
Infant (< 1 year)	24 (3.7)	8 (3.3)	16 (3.9)	
Pediatric (1–15 years)	65 (9.9)	22 (9.0)	43 (10.4)	
Adult (> 15 years)	438 (66.8)	131 (53.7)	307 (74.5)	
Unknown	129 (19.7)	83 (34.0)	46 (11.2)	
**Year of Transport**				0.53
2020	35 (5.3)	10 (4.1)	25 (6.1)	
2021	63 (9.6)	22 (9.0)	41 (9.9)	
2022	145 (22.1)	49 (20.1)	96 (23.3)	
2023	252 (38.4)	97 (39.7)	155 (37.6)	
2024	161 (24.5)	66 (27.1)	95 (23.1)	
**Type of mission**				< 0.01*
Primary mission	132 (20.1)	45 (18.4)	87 (21.1)	
Secondary mission	524 (79.9)	199 (81.6)	325 (78.9)	
**Time of Request**				0.22
08:00–12:00	224 (34.2)	42 (17.2)	182 (44.2)	
12:00–16:00	246 (37.5)	34 (13.9)	212 (51.5)	
16:00–20:00	39 (6.0)	21 (8.6)	18 (4.4)	
Unknown	147 (22.4)	147 (60.3)	0 (0)	
**Particulate Matter -** µg/m³, median (IQR)				
PM2.5	23 (14–41)	28 (19–49)	20 (13–35)	0.018*
PM10	38 (24–56)	40 (26–59)	36 (23–55)	0.40
**Origin**				0.07
Chiang Mai	377 (57.5)	157 (64.3)	220 (53.4)	
Chiang Rai	5 (0.8)	1 (0.4)	4 (1.0)	
Lampang	2 (0.3)	1 (0.4)	1 (0.2)	
Lamphun	1 (0.2)	1 (0.4)	0 (0)	
Mae Hong Son	265 (40.4)	80 (32.8)	185 (44.9)	
Nan	3 (0.5)	1 (0.4)	2 (0.5)	
Tak	2 (0.3)	2 (0.8)	0 (0)	
Unknown	1 (0.2)	1 (0.4)	0 (0)	
**Disease**				< 0.01*
STEMI	212 (32.3)	66 (27.05)	146 (35.44)	
Major Trauma	139 (21.2)	49 (20.1)	90 (21.8)	
Stroke	77 (11.7)	32 (13.1)	45 (10.9)	
Respiratory illnesses	81 (12.4)	34 (13.9)	47 (11.4)	
Cardiac emergencies	23 (3.5)	10 (4.1)	13 (3.2)	
GI emergencies	15 (2.3)	4 (1.6)	11 (2.7)	
OB-Gyn emergencies	29 (4.4)	14 (5.7)	15 (3.6)	
Neonatal emergencies	22 (3.4)	8 (3.3)	14 (3.4)	
Aortic emergencies	13 (2.0)	4 (1.6)	9 (2.2)	
Sepsis	19 (2.9)	3 (1.2)	16 (3.9)	
Neurology Emergencies	13 (1.9)	7 (2.9)	6 (1.4)	
Other	13 (2.0)	13 (5.3)	0 (0)	
**Survival at 24 h** ^†^	386 (58.8)	6 (2.5)	380 (92.2)	1.00
Death	30 (4.6)	1 (0.4)	29 (7.0)	
Unknown	240 (36.6)	237 (97.1)	3 (0.7)	
**Survival at 72 h** ^†^	373 (56.9)	5 (2.1)	368 (89.3)	0.96
Death	44 (6.7)	0 (0)	44 (10.7)	
unknown	244 (37.2)	239 (98.0)	5 (1.2)	

Abbreviations: IQR; Interquartile Range., Age group: Infant (< 1 year), Pediatric (1–15 years), Adult (> 15 years)., Significant results (*p* < 0.05) are marked with an asterisk (*). Remark: 2024 data cover January–September, †Survival denominators are based on available cases only (> 30% missing), data are exploratory and not part of the primary analysis

Among non-transported requests, leading non-transport categories were medical contraindication (63, 25.8%), lack of aircraft or landing-zone feasibility (58, 23.8%), adverse weather (46, 18.9%), and haze-related air pollution (31/244, 12.7%); inappropriate timing accounted for a further 31 (12.7%) show in Table 2. Medical contraindications were further detailed in Supplementary Table S1.

**Table 2:** Reasons for non-transport (Denial Reasons)

Reason Category	Cases (*n*)	Percent (%)
Medical contraindication	63	25.82%
Lack of aircraft / landing constraints	58	23.77%
Weather-related (Cloudy, storm, rain)	46	18.85%
Haze-related	31	12.70%
Inappropriate timing	31	12.70%
Poor prognosis	11	4.51%
Medical Team Issue	3	1.23%
No time benefit over ground transport	1	0.41%

Remark: Haze-related and weather-related non-transport were mutually exclusive; in this dataset, days with precipitation/storms did not overlap with haze events; inappropriate timing includes twilight hours, crew duty-time limits, overlapping missions, or curfew

**Fig. 1:**
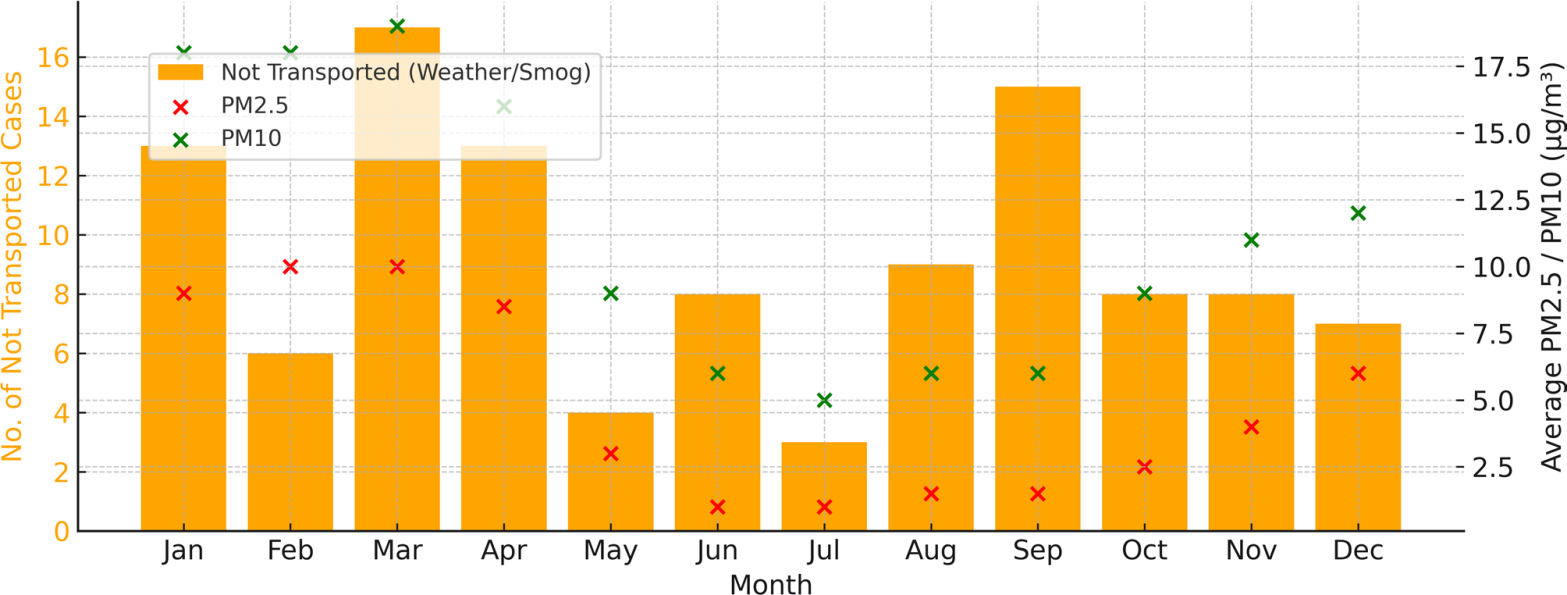
Monthly distribution of air medical transport cases that were not transported due to weather or haze (bars, left y-axis), overlaid with the monthly average concentrations of PM2.5 *(µg/m³)* (red ×) and PM10 *(µg/m³)* (green ×) from 2020 to 2024 (right y-axis). A seasonal peak in non-transported cases is observed in March, coinciding with the highest average PM concentrations. This suggests a potential environmental impact on air medical transport availability; 2024 data represent January–September only

In multivariable logistic regression (Table 3), three factors were independently associated with transport completion. Requests during twilight hours (16:00–20:00) were less likely to be transport (adjusted OR 0.29, 95% CI 0.13–0.67; *p* < 0.01). Secondary missions were more likely to be transport than primary missions (adjusted OR 3.51, 1.47–8.40; *p* < 0.01). Increasing PM2.5 concentration was associated with lower odds of transport (adjusted OR 0.99, 0.97–0.99; *p* = 0.018). PM10 was not associated with transport (*p* = 0.40). Sensitivity analyses excluding cases with missing values yielded results consistent with the primary model. Seasonal patterns were evident: non-transport rose between February and April, peaking in March, coincident with higher ambient PM2.5 and PM10 concentrations (Fig. 1).

**Table 3:** Logistic regression analysis of factors associated with successful operation of air medical transport

Parameter	Univariable			Multivariable		
	OR	95% CI	*p*-value	OR	95% CI	*p*-value
**Demographic**						
Male (ref: Female)	1.54	0.66–3.60	0.32			
Age group Child (ref: Adult)	0.33	0.05–2.00	0.23			
Age group Infant (ref: Adult)	0.33	0.05–2.00	0.23			
**Diagnosis group** (ref: other diagnoses)						
Sepsis	0.46	0.15–1.37	0.16	0.53	0.14–2.07	0.36
STEMI	0.63	0.36–1.08	0.10	0.96	0.49–1.89	0.91
**Secondary Mission** (ref: Primary)	2.79	1.72–4.54	< 0.01*	3.51	1.47–8.40	< 0.01*
**Time of request** (ref: 8:00–12:00)						
16:00–20:00	0.20	0.10–0.40	< 0.01*	0.29	0.13–0.67	< 0.01*
**Other reasons**						
Poor prognosis	0.23	0.05–1.16	0.07	0.18	0.02–2.08	0.17
PM2.5	0.99	0.97–0.99	0.01*	0.99	0.97–0.99	0.018*
PM10	1.05	0.94–1.17	0.40			

**Abbreviations**: OR = Odds Ratio; CI = Confidence Interval; PM2.5 = Particulate Matter ≤ 2.5 μm; PM10 = Particulate Matter ≤ 10 μm., Ref = Reference, Significant results (*p* < 0.05) are marked with an asterisk (*)

## Discussion

This study highlights the multifactorial challenges affecting AMS in a resource-constrained, mountainous region of Northern Thailand. Among all AMT requests approximately one-third were not transported. The leading causes included medical contraindications, limited aircraft or landing zone constraints, adverse weather, and air pollution, particularly seasonal haze. These findings reflect a broader pattern observed in rural and low-resource settings, where patient needs must be balanced against logistical readiness, infrastructure limitations, and environmental hazards [14].

One of the most striking findings was the high frequency of non-transport due to aircraft unavailability or landing zone. In Thailand, air medical missions are largely dependent on shared resources from the military, police, or governmental agencies, all of which may be unavailable at critical moments due to competing priorities. This contrasts with high-resource settings such as the United States, Germany, and parts of Japan, where dedicated, 24/7 HEMS fleets exist [1, 11]. Compounding this issue is the limited helipad infrastructure, particularly in remote or highland communities. While regional Aeromedical Dispatch Center have made progress by investing in new helipads, development remains slow due to challenges in terrain assessment, land-use negotiation, and first-responder training.

Weather-related and visibility issues were also among the most common reasons for non-transport. Similar to studies conducted in Alaska [15], Australia [16], and Canada [17], our data confirm that poor weather, including storms, cloud cover, and low light conditions, can frequently prevent safe takeoff or landing, even when aircraft are available. A notable concentration of denied flights occurred between 16:00 and 20:00, corresponding with twilight hours when visual flight becomes increasingly hazardous [18]. In our cohort, adverse weather alone accounted for nearly one-fifth of denials, underscoring its importance alongside air pollution as a key environmental barrier. Moreover, this figure likely underestimates the true impact of weather, because during rainfall or storms, flight infeasibility is often presumed and requests for HEMS may not be initiated at all. This high frequency underscores that weather remains a major environmental barrier, second only to air pollution, in limiting air medical transport feasibility.

PM2.5, or particulate matter with aerodynamic diameters less than 2.5 microns, poses a critical challenge to helicopter operations, especially in regions reliant on visual flight rules (VFR) such as Northern Thailand [19]. Unlike PM10, PM2.5 particles scatter visible light more efficiently, remain airborne for longer durations, and readily interact with water vapor to form dense haze, thereby reducing visibility below safe operating thresholds [20, 21]. These ultrafine particles primarily originate from biomass burning, vehicle emissions, and industrial processes, with seasonal surges driven by agricultural fires and transboundary pollution [22]. During haze seasons, visibility often drops below 3 km, and cloud ceilings fall beneath 1,000 feet [23]. These environmental conditions can restrict helicopter takeoffs, landings, and operations, especially in mountainous or urban terrain, increasing the likelihood of spatial disorientation and mission cancellations. In the SkyDoc program, AMT missions in this region are frequently withheld when the AQI exceeds 150, which is often a threshold communicated during morning briefings. This has led to some cases being excluded from coordination altogether due to the presumed infeasibility of flight. As a result, the impact of PM2.5 may be underreported in existing data, and its association with non-transport could be even stronger than shown. A particularly vivid incident occurred during the 2015 Southeast Asian haze crisis, when elevated PM2.5 levels grounded both commercial and emergency flights across the region [21, 24–26].

Globally, AMT systems face a wide spectrum of operational and environmental barriers, though the specific factors vary by geography. For instance, in Latin America, Araiza et al. highlighted that high-altitude conditions and weather-induced risks, compounded by insufficient helipad infrastructure, often impair operational efficiency [7]. Similarly, in resource-limited settings, Johnson and Luscombe pointed to delays in dispatch and coordination gaps as major impediments to timely helicopter response [12]. In India, Singh et al. noted that terrain and extreme altitude presented physical challenges to AMT, but did not report fine particulate air pollution as a significant factor [11]. Studies in Alaska and Taiwan also reflect this, identifying harsh weather conditions such as snow, wind, and typhoons as the principal causes of flight delays or cancellations [13]. Our study, however, reveals an additional and largely underrepresented environmental constraint: seasonal exposure to PM2.5. Unlike the broader meteorological variables emphasized in previous literature, fine particulate air pollution in Northern Thailand appears to play a unique role by simultaneously obstructing flight safety and escalating demand for emergency medical services. For example, during haze season, elevated PM2.5 not only limits visibility and cancels missions, but also leads to spikes in emergency department visits for acute coronary syndromes and pneumonia [27]. This dual burden limited aerial access and increased medical need, which amplifies the vulnerability of populations in remote areas, underscoring the importance of geographically tailored mitigation strategies that account for the health and operational impacts of air pollution. In light of these findings, strengthening AMS resilience in Northern Thailand calls for a coordinated, real-time approach. Building on recent steps that include hourly PM2.5 reporting and route-specific forecasts developed with the Northern Meteorological Center, a national strategy should deploy dedicated Instrument Flight Rules (IFR)-capable fleets for low-visibility operations [28], use GIS to target helipad expansion in underserved terrain [29], and integrate meteorology, air-quality, and aircraft-readiness data into a single dispatch dashboard to streamline mission triage. Haze-adapted flight protocols and IFR training for pilots and dispatchers should be standard, while broader environmental measures remain necessary because seasonal PM2.5 both degrades aviation safety and drives spikes in acute cardiovascular and respiratory illness [27]. When flight paths cannot avoid haze, crew-safety protocols should include in-flight air-quality monitoring, appropriate protective measures, and timely early warnings.

Additionally, in this study, medical contraindication was the most frequent reason for AMT non-transport. These included patients with non-time-sensitive conditions, or contraindications such as active tuberculosis, COVID-19, untreated pneumothorax, pneumocephalus, or those considered too sick or expectant. Similar findings have been reported. In the Netherlands, over half of HEMS missions were cancelled due to patient reassessment, which was found to be undertriage [30], while in Canada, 76% were cancelled due to not meeting trauma criteria [31]. Integrating clinical triage tools with real-time environmental data can enhance decision accuracy, ensuring both patient safety and operational efficiency. Future policy should support the development of dynamic protocols that account for both medical and environmental conditions, particularly in resource-limited or high-risk settings.

This study has several limitations. First, as a retrospective study several variables had missing values (e.g., gender, age, time of request), which may reduce representativeness, although sensitivity analyses produced consistent results. Notably, time-of-request missingness clustered in the non-transported group (60.3%). This non-random missingness could distort the observed twilight association; although sensitivity analyses were consistent, readers should interpret this finding with appropriate caution. Second, air quality data were assigned from the nearest monitoring stations and PM2.5 was used as a proxy for visibility in the absence of METAR or VFR/IFR data, so overlap with other weather phenomena cannot be excluded. Third, although PM2.5 was independently associated with overall mission outcome, we were unable to model haze-related denials separately due to the small sample size. Moreover, the observed number of haze-related denials likely underestimates the true effect of air pollution, because during severe haze some requests may never have been initiated at all when flight infeasibility was presumed. This pre-request suppression would bias our estimates toward the null, suggesting that the actual impact of PM2.5 on mission feasibility may be even stronger than observed. Fourth, pandemic-related constraints were not analyzed separately and were coded under broader categories. Fifth, downstream clinical outcomes and survival data (> 30% missing) were not fully assessed and should be interpreted as exploratory.

Future research should adopt prospective designs with real-time data capture to minimize missingness and better characterize mission feasibility. System-level information such as daily call volumes, ground transfers, and organizational responses should be incorporated to clarify the denominator of need during haze seasons. Integrating aeronautical visibility metrics (e.g., METAR reports, VFR/IFR status) with spatio-temporal air pollution models may improve exposure assessment. Finally, linking mission outcomes with downstream clinical outcomes will provide a more comprehensive understanding of the impact of non-transport on patient care.

## Conclusion

AMT in Northern Thailand is significantly hindered by a combination of environmental hazards, limited infrastructure, and operational constraints. Seasonal haze and elevated PM2.5 levels emerged as key predictors of non-transport, along with twilight-hour mission requests and limited aircraft availability. Improving equitable access to AMT in this region requires a coordinated strategy that includes environmental surveillance, dedicated air fleets with IFR capability, infrastructure development, and policy reform. Addressing these barriers is essential not only for enhancing emergency care access, but also for building a resilient and environmentally adaptive emergency medical system.

## Supplementary Information

Below is the link to the electronic supplementary material.

Supplementary Material 1

## Data Availability

The datasets are available from the corresponding author upon reasonable request.

## Declarations

### Patient and public involvement

Patient and public involvement was integral to the design, execution, and dissemination of this study. In recognition of the importance of the incorporation of patient perspectives and to ensure the relevance of our research, we engaged with patients and members of the public at multiple stages of the research process.
