# Supplementary material for: Barriers to helicopter emergency medical services in a Haze-Prone, mountainous region of Northern Thailand
Source: Scand J Trauma Resusc Emerg Med. 2025 Nov 13;33:182. doi: 10.1186/s13049-025-01498-w (PMC12613902; doi:10.1186/s13049-025-01498-w)
Supplement: Supplementary file 1 — Supplementary Material 1 [file 13049_2025_1498_MOESM1_ESM.docx]

Supplementary

**Table S 1 Distribution of non-transport by Air Medical Due to Medical Contraindications**

| **Medical Contraindication** | **Cases (n)** | **Percent (%)** |
| --- | --- | --- |
| Confirmed COVID-19 infection | 19 | 30.2% |
| Non–time-sensitive conditions | 14 | 22.2% |
| Too sick/expectant prognosis | 14 | 22.2% |
| Other spreading infection | 7 | 11.1% |
| Active/suspected tuberculosis | 3 | 4.8% |
| Pneumocephalus | 3 | 4.8% |
| Untreated pneumothorax | 2 | 3.2% |
| Other | 1 | 1.6% |
| **Total** | 63 | 100% |
